# Introducing best practice for reproducibility in government

**DOI:** 10.23889/ijpds.v8i2.2323

**Published:** 2023-09-14

**Authors:** Megan Munro, Patrycja Delong-Smith, Pete Matthews, Loes Charlton, Megan Lyons, James Tucker

**Affiliations:** 1 Office for National Statistics, Newport, United Kingdom

There is an appetite to improve the reproducibility of quantitative analysis undertaken across government. Our team supports conversion of regular publications into Reproducible Analytical Pipelines (RAP). To achieve this, we developed a roadmap to work with analysts in our organisation and help them transform their pipelines and build their skills.

An audit of the current RAP status of all regular pipelines assisted in resource allocation and planning. The maturity of a RAP is evaluated on 7 criteria required to reach a minimum viable product (MVP) and 7 additional advanced criteria as outlined by the Analysis Function (AF). We use a combination of hands-on pair-coding with analysis teams, regular and ad hoc code reviews, and training sessions to convert existing pipelines into RAPs, while simultaneously upskilling the analysts. We have also developed guidance and training documentation to share internally and externally.

Currently, out of 73 regular publications, 12 have reached the MVP, with an average score of 4.36 out of 7. This scoring is reassessed monthly, allowing us to track the progress in real-time. Self-assessment of technical skills increased by between 43% and 89% and 97% said their understanding of RAP principals improved because of the training and 77% said they are now able to implement best practice into their work. By working with the pipeline owners instead of just refactoring the code directly, we are ensuring business resilience. The in-depth knowledge of the pipeline and skills required to maintain it are present within the analysis team. Publishing our methods, documentation and tools facilitates adoption of RAP for those without a dedicated RAP team.

We are on track to convert all our regular publications into RAPs and move to “RAP by default”, in line with the AF RAP Strategy. This will improve the reproducibility, quality, efficiency, transparency, and trustworthiness of analysis within government. We hope other organisations can learn from our methods.

